# Mr. Atkinson, on Apoplexy

**Published:** 1802-02

**Authors:** James Atkinson

**Affiliations:** Abingdon


### *To the Editors of the Medical and Phyfical Journal.*

GENTLEMEN,

ON looking over your Journal for December 1801, I find a Statement of a Cafe of Apoplexy by Mr. Crowfoot, and copioufly anfwered by Dr. Langflow. I have taken the liberty of addreffing to you the following Obfervations, as they occurred on the perufal of that Anfwer; and as I conceive your ineftimable Work is intended for the free and candid inveftigation of whatever relates to the caufe, treatment, or nature of difeafes, any apology would be unneceffary.

To afcertain any fact in medicine, and even furgery, owing to the various theories and opinions of mankind, has always been attended with much previous difcuffion. The fcience is ftill

---

ſtill imperfect, and appearances on diſſection too often only tend to prove the futility of human comprehenſion; for when there is every ſymptom of diſeaſed viſcera, how frequently does it appear to Anatomiſts, that the very part has ſuffered no derangement in its ſtructure, or has been affected in the manner the ſymptoms might naturally indicate. The moſt profound Phyſicians are never certain that their concluſions are juſt. When indulging in the enthuſiaſm of meditation, on abſorbents, excitability, or the nervous power, they are apt to forget that the human frame is the object of their inquiry; they are carried into a maze of theory and conjecture, and turn back, aſtoniſhed at the fertility of imagination and the train of reaſoning they have been led to adopt. There ſtill hangs a myſtery over many points in the animal œconomy, which puzzles the moſt acute and diſcerning to penetrate. However, there is little conſolation in being perſuaded of the incompleteneſs and uncertainty of medical knowledge; yet every ſuggeſtion or attempt for the improvement of ſcience is laudable, and cannot be unacceptable to the profeſſion. I would not wiſh to inſinuate by theſe deſultory Remarks, that what I have to advance at preſent is new or particularly important. In the Caſe of Apoplexy above alluded to, Dr. Langſlow has been very active and eloquent, to prove that the proximate cauſe of that diſeaſe is extravaſation upon the brain. I am neither acquainted with him nor Mr. Crowfoot perſonally, and I ſpeak without prejudice. He appears to me a gentleman of reſpectable acquirements, and I truſt he will hear my critique with impartiality, and without offence. Liberality of ſentiment in profeſſional inquiries certainly ought to be allowed, and every private conſideration generouſly ſacrificed to public utility.

In regard to the efforts of Nature, I do believe with Doctor Langſlow, that the *vis medicatrix naturæ* is the moſt powerful agent; that the feeble aſſiſtance of many medical practitioners can claim little praiſe; and that this agent alone, frequently expels the morbific diatheſis from the ſyſtem, in ſpite of medicine and diſeaſe. What I ſhall offer to Dr. Langſlow's conſideration is briefly this: Had there been extravaſation in Mr. Crowfoot's caſe, is it poſſible that it could be abſorbed, though after profuſe bleeding, ſo as to produce an *immediate* mitigation of every diſtreſſing ſymptom? Does not the ſucceſs of ſuch practice anſwer better to the ſuppoſition, for it can only be ſuppoſition, that there was no extravaſation, only an increaſed determination to the head, rendering the coats of the arteries turgid, and by their preſſure on the medullary portion of the brain, cauſing ſtupor, coma, or delirium? And may not the diſtended carotids, preſſing againſt the correſponding or accompanying nerves nerves which ſupply the ſtomach, be the cauſe of the ſtomachic affection? If Apoplexy be invariably the conſequence of compreſſion of the brain, the morbid increaſed pulſation of the arteries will cauſe that compreſſion. Extravaſation, exudation, or effuſion, does not neceſſarily conſtitute compreſſion. The ſubſtance of the brain is known to be the moſt irritable part of our body, and the leaſt ſtimulus from the extreme veſſels will be the ſame, and attended by the ſame conſequences, as compreſſion; therefore, there may be compreſſion, without extravaſation, exudation, or effuſion. And although extravaſation does produce Apoplexy, may not genuine Apoplexy be produced without extravaſation? A patient with contuſio cranii will have Apoplexy when extravaſation has evidently taken place; but when another patient falls down apoplectic,* if a total abolition of the ſenſes, ſonorous breathing, the eyes vacant, and a ſpaſmodic fall of the jaw, may be allowed ſymptoms of Apoplexy; I ſay, can it be inſiſted on, that this patient had extravaſation when, *immediately* after the paroxyſm, he is as well as before the attack? Could the lymphatics of the brain, or any other power, abſorb the effuſion in ſo ſhort a time?

With reſpect to the propriety of exhibiting emetics in Apoplexy, I think the action of vomiting muſt aggravate the diſeaſe: topical applications, by diminiſhing the energy of the veſſels in the head, without debilitating the ſyſtem, muſt be eminently uſeful. In this the ideas of Dr. Langſlow are certainly correct; but when he poſitively aſſerts, "whenever Apoplexy occurs, and there is no fracture or tumour, that extravaſation, exudation, or effuſion upon the brain *always does exiſt*," I would aſk, has his impetuoſity led him to do much credit to *his* profeſſional character? for it is moſt probable that of ten caſes of Apoplexy, which are not fatal, not above one or two is cauſed by extravaſation of blood upon the brain. The following recent caſe may ſerve as an illuſtration.

Elizabeth Trinder, of Shippon, a poor girl, ſeventeen years of age, of a delicate habit, whoſe mother died of typhus † a few

* Or as Boerhaave, nearly two hundred years ago, defined it: "Quando repente actio quinque ſenſuum externorum, tum internorum, omneſque motus voluntarii abolentur, ſuperſtite pulſu plerumque forti, et reſpiratione difficili, magna, ſtertente, una cum imagine profundi perpetuique ſomni."

Boerh. Aphor. 1008.

† Amongſt the various miſeries of which war and its concomitant evils are productive, the late ſcarcity has been the moſt ſeverely felt, and has been particularly fatal to numbers of the poorer claſſes of ſociety. Penury is the ſource of many diſeaſes, very obſtinate and alarming in their attack.

few days before, was attacked with pyrexia on the 28th of November laſt. She continued in a weak, debilitated ſtate, with great depreſſion of ſpirits, till early in December, when ſhe experienced the more violent ſymptoms of Apoplexy. I ſaw her in this alarming ſituation; her eyes were half cloſed, the ſurface of her whole body was pale, and cold as death, and ſhe paſſed her fæces and urine involuntarily. In this ſtate ſhe laid nearly two days inſenſible. Sometimes, ſhe with difficulty lifted her hand to her head, and then dropped it as it were, lifeleſs on the pillow, muttering at the ſame time, "O my head, my head." Her reſpiration made a ſnorting noiſe, and ſhe ſeemed haſtening to diſſolution. After much fruitleſs remonſtrance, the family being perſuaded that nothing could ſave her, they reluctantly allowed a large bliſter to be applied below the occiput, on the morning of the 5th of December, and in the evening two others were applied to the arms. It is remarkable, that in a few hours afterwards ſhe raiſed her eyes, and ſpoke rationally. The pain in her head was conſiderably relieved. She had now ſome appetite, and ſince then her health gradually improved, and ſhe has had few febrile paroxyſms.

Dr. Langſlow, I ſuppoſe, would not heſitate to pronounce, whether this was ſanguineous or ſerous Apoplexy, and if there was in this caſe extravaſation, exudation, or effuſion. If there was an effuſion, whether it was of blood or ſerum upon the brain, could it be ſo ſpeedily abſorbed? My concluſion is this, that there often occurs Apoplexy without an effuſion or exudation of blood or ſerum upon the brain; and, that when there is *conſiderable* extravaſations, the caſe is generally fatal. When there is extravaſation, the patient's recovery muſt be ſlow and difficult; for the power of abſorption cannot be ſo quick as Dr. Langſlow has imagined. When the patient's recovery is *immediate*, on diminiſhed excitement, I think it preſumptive evidence, that there has been no extravaſation, exudation, or effuſion. The above caſe may ſtand as a confirmation

The conſtitution being weakened, by receiving little nouriſhment, is unable to bear accumulating complaints, and ſinks exhauſted. For when any epidemic, or other diſeaſe, is introduced into the houſes of the ſtarving poor, medicines are often of little avail. They want not only the comforts, but what is, even in health, abſolutely neceſſary to exiſtence. In Abingdon, the fatality of the typhus fever, amongſt the poor, has been very remarkable, and generally attended with acute delirium. To the celebrated Beddoes's queſtion, Whether children born during the ſcarcity, are leſs healthy than thoſe born under other circumſtances, or prior to that diſtreſsful period? it may be ſaid, that his fears are unfortunately too well grounded.

firmation of the good effects of blisters, applied when there is the least hope of their being succefsful.

I cannot conclude this paper, without observing the dignity and importance which some attach to a physician; I mean particularly in the country. But common judgment is generally directed to the supposed rank a person holds in society, and not to his individual merits. A surgeon enjoys the respectability which follows the exercise of that department of the profession, let his abilities be great or indifferent; but to regard a man according to the endowments of his mind, seems to me most rational. It becomes no sensible man to be ambitious of a long etcetera of fellowships and titles; they will make him not one atom the wiser. It is, alas! the diploma splendors of the college, which infatuate the multitude; but when divested of these advantages, the charm is destroyed. There is a modesty in controversy which enhances merit; but Dr. Langslow assumes a confidence and a pride, when speaking of his own skill, which is little respectful. There is a delicate and a splendid way of writing on such subjects. When disputes become warm and ill-natured, they cease to be useful. Men of talents have a right to be liberal and just; but their charter does not extend so far, as to allow them to be contemptuous.

I am, &c.

*Abingdon, Jan.* 1, 1802. JAMES ATKINSON.

